# Neighborhood-level social vulnerability and individual-level cognitive and motor functioning over time in older non-Latino Black and Latino adults

**DOI:** 10.3389/fnhum.2023.1125906

**Published:** 2023-05-12

**Authors:** Melissa Lamar, Kiarri N. Kershaw, Sue E. Leurgans, R. Reshmi Mukherjee, Brittney S. Lange-Maia, David X. Marquez, Lisa L. Barnes

**Affiliations:** ^1^Rush Alzheimer’s Disease Center, Rush University Medical Center, Chicago, IL, United States; ^2^Department of Psychiatry and Behavioral Sciences, Rush University Medical Center, Chicago, IL, United States; ^3^Department of Preventive Medicine, Northwestern University Feinberg School of Medicine, Chicago, IL, United States; ^4^Department of Neurological Sciences, Rush University Medical Center, Chicago, IL, United States; ^5^Claremont McKenna College, Claremont, CA, United States; ^6^Department of Family and Preventive Medicine, Rush University Medical Center, Chicago, IL, United States; ^7^Department of Kinesiology and Nutrition, University of Illinois Chicago, Chicago, IL, United States

**Keywords:** neighborhood vulnerability, social vulnerability, cognition, motor functioning, non-Latino Black adults, Latinos, aging, African Americans

## Abstract

**Introduction:**

Despite known health disparities in cognitive aging, a comprehensive rationale for the increased burden in older minoritized populations including non-Latino Black and Latino adults has yet to be elucidated. While most work has focused on person-specific risk, studies are increasingly assessing neighborhood-level risk. We evaluated multiple aspects of the environmental milieu that may be critical when considering vulnerability to adverse health outcomes.

**Methods:**

We investigated associations between a Census-tract derived Social Vulnerability Index (SVI) and level of and change in cognitive and motor functioning in 780 older adults (590 non-Latino Black adults, ∼73 years old at baseline; 190 Latinos, ∼70 years old baseline). Total SVI scores (higher = greater neighborhood-level vulnerability) were combined with annual evaluations of cognitive and motor functioning (follow-up ranged from 2 to 18 years). Demographically-adjusted mixed linear regression models tested for associations between SVI and cognitive and motor outcomes in analyses stratified by ethno-racial group.

**Results:**

For non-Latino Black participants, higher SVI scores were associated with lower levels of global cognitive and motor functioning-specifically, episodic memory, motor dexterity and gait-as well as longitudinal change in visuospatial abilities and hand strength. For Latinos, higher SVI scores were associated with lower levels of global motor functioning only-specifically, motor dexterity; there were no significant associations between SVI and change in motor functioning.

**Discussion:**

Neighborhood-level social vulnerability is associated with cognitive and motor functioning in non-Latino Black and Latino older adults, although associations appear to contribute to level more so than longitudinal change.

## 1. Introduction

Despite known health disparities in risk for and development of Alzheimer’s disease and related dementias (ADRD) in older minoritized populations including non-Latino Black and Latino adults (Matthews et al., 2019), a comprehensive evidence-based explanation for this increased burden has yet to be elucidated. Furthermore, to date, most work investigating health disparities in cognitive aging and ADRD have focused on person-specific factors (e.g., De Anda-Duran et al., 2022), with less work investigating neighborhood-level vulnerabilities that may contribute to increased risk of ADRD within minoritized communities. While burgeoning work increasingly suggests that specific aspects of the neighborhood milieu in which a person lives, including higher crime (Ruiz et al., 2021), residential segregation including neighborhood ethnic density (Pohl et al., 2021), and urban overcrowding (Besser et al., 2018), individually contribute to adverse cognitive aging, much of this work has been conducted within combined ethno-racial analytic samples with or without comparison to non-Latino White adults. While some studies note more robust relationships between adverse neighborhood features (Besser et al., 2018; Pohl et al., 2021) and negative cognitive outcomes for minoritized participants compared to non-Latino White participants, there are known differences in lived experience between minoritized ethno-racial groups living in the US (e.g., Lamar et al., 2020, 2021), which may also include differences in their respective neighborhood milieu. Thus, if we are to fully understand whether and how neighborhood-level factors associate with cognitive aging trajectories among specific ethno-racial groups, studies should focus on within group heterogeneity using comprehensive neighborhood-level evaluations.

Using longitudinal data from nearly 800 non-Latino Black and Latino participants of the Rush Alzheimer’s Disease Center (RADC) Minority Aging Research Study and the Latino Core, respectively, we evaluated neighborhood-level vulnerability using the Social Vulnerability Index (SVI) and investigated its associations with participant-level global and domain-specific cognitive and motor functioning within each ethno-racial group. The SVI is a comprehensive evaluation of 15 social factors including minority status and language that was created by the Centers for Disease Control and Prevention (CDC) (CDC, 2000). While it and the Area Deprivation Index (Kind et al., 2014) have been recommended by several government agencies to assist with the equitable allocation of resources (e.g., the COVID-19 vaccine; National Academies of Sciences, Engineering, and Medicine, 2020), only the SVI takes into consideration neighborhood-level ethno-racial make-up, a factor previously associated with individual-level cognition (Pohl et al., 2021), as well as health-related risk factors for adverse cognitive aging (e.g., incident hypertension; Gao et al., 2022). We hypothesized that higher SVI levels would be associated with lower levels of and faster declines in cognition and motor functioning with potentially differential involvement of cognitive and motor domains by ethno-racial group.

## 2. Materials and methods

### 2.1. Participants

Participants were self-identified non-Latino Black or Latino adults 60 years or older, enrolled in either the Minority Aging Research Study (MARS: 2004 to present) (Barnes et al., 2012b), or the RADC Latino Core (LATC; 2015 to present) (Marquez et al., 2020), both ongoing longitudinal cohort studies of aging. Participants in these cohorts are recruited from a variety of community-based settings that cater to minoritized seniors in the metropolitan Chicago area and outlying suburbs; enrollment is ongoing and requires that older adults enroll free of known dementia at baseline and agree to annual, in-home, evaluations. These studies are identical in essential details including a harmonized protocol that contains the same cognitive and motor measures and is conducted by the same investigators with a single population studies team. The Institutional Review Board of Rush University Medical Center approved these studies and participants gave written informed consent in accordance with the Declaration of Helsinki.

We excluded participants diagnosed with dementia at baseline using a uniform structured clinical evaluation (Bennett et al., 2006) and NINDS/ADRDA criteria (McKhann et al., 1984). Only those non-Latino Black and Latino participants who completed a baseline evaluation and provided a valid address for geocoding purposes were included in this study. At the time of these analyses, 780 participants (590 non-Latino Black Americans and 190 Latinos) met all study eligibility criteria including >2 annual evaluations and thus contributed to our analytic sample. Please note, to maximize our sample and remain consistent for participant inclusion across cognitive and motor outcomes, we required >2 annual cognitive but not motor evaluations. Thus, non-Latino Black participants had a mean of 7.8 ± 3.9 annual visits (range = 2–18) for cognitive data, and 6.2 ± 3.6 (range = 0–16) for motor data; Latinos had a mean of 4.4 ± 1.2 annual visits (range = 2–7) for cognitive data and 3.0 ± 1.0 (range = 1–5) for motor data.

### 2.2. Geocoding participant addresses

Participants provided their current address at study entry or shortly thereafter; because all testing is traditionally done face-to-face within participants’ homes, they were queried for their current address at each subsequent visit to ensure accuracy and/or document changes in residential location. Participants’ addresses were reviewed and corrected for clerical errors prior to conducting internal geocoding using geographic information systems (GIS) mapping software ESRI ArcGIS and US Census TigerLine data (i.e., 2000, 2010, 2020; United States Bureau of the Census, 2020). The analytic baseline for projects involving GIS-related data was the first study visit that corresponded to the first geocoded address.

We documented individual participant duration of exposure to their neighborhood environment moving forward in time, starting with their analytic baseline year. Within the Minority Aging Research Study (MARS), 168 participants (20% of the entire MARS study cohort, *n* = 802 at the time of geocoding) reported a change in address over the course of their study participation. For Latino Core (LATC), 37 participants (15% of the entire LATC study cohort, *n* = 245 at the time of geocoding) reported an address change over the course of their study participation. Thus, while we do not know retrospective, i.e., historic, duration of exposure prior to study entry (having not asked all participants how long they had lived at their initial address), based on the fact that the majority of participants in MARS (80%) and LATC (85%) lived at their initially named addresses throughout the entire course of their study participation, we are relatively confident that historic duration of exposure is similar to our documented prospective duration and suggests high residential stability for the majority of participants.

### 2.3. Social vulnerability index

The Social Vulnerability Index (SVI; CDC, 2000), ranks US Census tracts based on 15 social factors that are categorized into four themes. These four themes and the indices that comprised them were: (1) socioeconomic status: below 150% poverty, unemployment, housing cost burden, no high school diploma, no health insurance; (2) household characteristics: aged 65 and older, aged 17 or younger, those with disabilities, single-parent household; (3), minority status and language: racial and ethnic residential categorizations, aged 5 or older who speaks English less than well; and (4) housing and transportation: multi-unit structure, mobile homes, crowding, group quarters, and no vehicle. Participants’ analytic baseline year was used to determine the SVI year of data used for determination of their total score (e.g., 2017 and 2018 analytic baseline years employed 2018 SVI data). The total SVI score ranged from 0.00 (least vulnerable) to 1.00 (most vulnerable) with a higher score reflecting a more vulnerable Census tract for a given participant’s address.

Traditionally used by the Centers for Disease Control and Prevention (CDC) to quantify vulnerability to human suffering and financial loss in the event of disaster (Flanagan et al., 2011) including the recent COVID-19 pandemic (Ong and Ong, 2020), the SVI has been increasingly applied to more personal health-related variables (e.g., physical activity levels; An and Xiang, 2015) including those specific to older adults (e.g., frailty Armstrong et al., 2015). Furthermore, the SVI measures many of the same social factors highlighted as critical when considering the concept of vulnerability related to health disparities research generally (Grabovschi et al., 2013), and within aging research more specifically (Hill et al., 2015).

### 2.4. Cognitive and motor function

All participants underwent a cognitive evaluation administered in an identical fashion at annual evaluations (Barnes et al., 2012b; Marquez et al., 2020). Nineteen tests assessed the following five cognitive domains: episodic memory (two immediate and delayed story recall tests; word list memory, recall and recognition), semantic memory (confrontation naming; word reading; verbal fluency), working memory (digit forward and backward span; digit ordering), perceptual speed (Stroop subtests; symbol digit modality; number comparisons), and visuospatial ability (line orientation; progressive matrices). Raw scores were converted to standard z-scores using the baseline mean (SD) of the entire cohort, and the z-scores of all tests for each domain were then averaged for the five cognitive domains. A global cognitive function score was also derived averaging a person’s standard scores across all 19 test scores. Psychometric information on these summary scores has been deemed adequate (e.g., Barnes et al., 2012a).

Participants were also given 10 motor performance tests. As outlined in detail elsewhere (Buchman et al., 2019), manual (i.e., hand) strength was evaluated via grip and pinch, measured bilaterally and computed separately, using the Jamar hydraulic hand dynamometer (Lafayette Instruments, Lafeyette, IN, USA). Upper extremity dexterity was based on the average of four trials (2 right, 2 left) of finger tapping registered via the index finger using an electronic tapper device (Western Psychological Services, Los Angeles, CA, USA) as well as successful Purdue Pegboard placement. Gait was evaluated by the time (in seconds) and the number of steps taken to execute an eight foot walk and 360° turn, respectively. Balance was also measured through leg and toe stand tasks. All 10 measures were scaled and averaged to obtain a global motor functioning score and three motor domains (hand strength, dexterity, and gait) as outlined above and previously validated (Buchman et al., 2011).

### 2.5. Statistical analysis

Descriptive summaries of all variables were conducted as were quality checks of the baseline SVI data including histograms and QQ plots to determine normality of the data (it was deemed adequate). Linear mixed effects models were used to assess the relationship between baseline Social Vulnerability Index (SVI) (total score) and level of and longitudinal change in global cognition and global motor functioning as separate outcomes. Additional terms in the model included age, sex, education, and interactions of each of these variables with time (in study). We followed up these analyses with an investigation of the five cognitive domain scores and the three motor domain scores. As previously stated, given differences in the lived experience of non-Latino Black and Latino adults living in the US (e.g., Lamar et al., 2020, 2021), we conducted analyses stratified by ethno-racial group, i.e., separately for non-Latino Black adults and Latinos. An additional series of analyses conducted within Latinos added terms for language preference of testing (Spanish versus English) and its interaction with time (Latino Model 2) given that language preference may impact cognitive trajectories not only on its own, but also serve as a proxy for the large positive increase in test scores often seen by older Spanish-speaking Latinos after their initial exposure to cognitive testing (Early et al., 2013). All analyses were conducted using SAS/STAT software, Version 9.4 (SAS Institute, Cary, NC, USA); significance was set at *p* < 0.05.

## 3. Results

**FIGURE 1:**
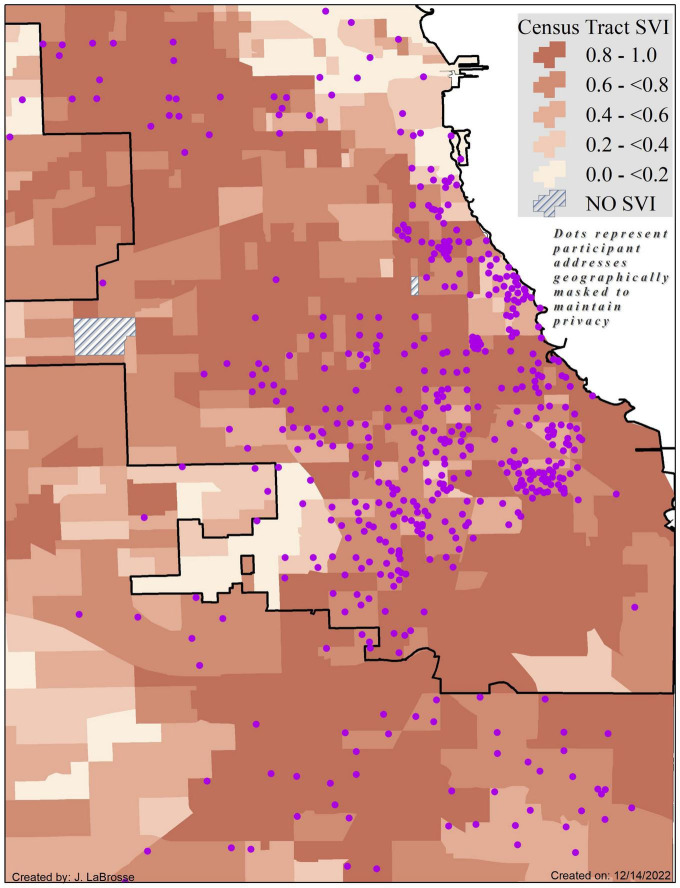
Census tract level Social Vulnerability Index scores by quintiles for the Chicagoland area as measured by the Centers for Disease Control and Prevention with non-Latino Black participant locations (purple dots) geographically masked to protect confidentiality and maintain privacy. Higher SVI scores and corresponding darker shades of orange signify greater vulnerability. Map coverage is reflective of the geographic center of participant addresses.

**FIGURE 2:**
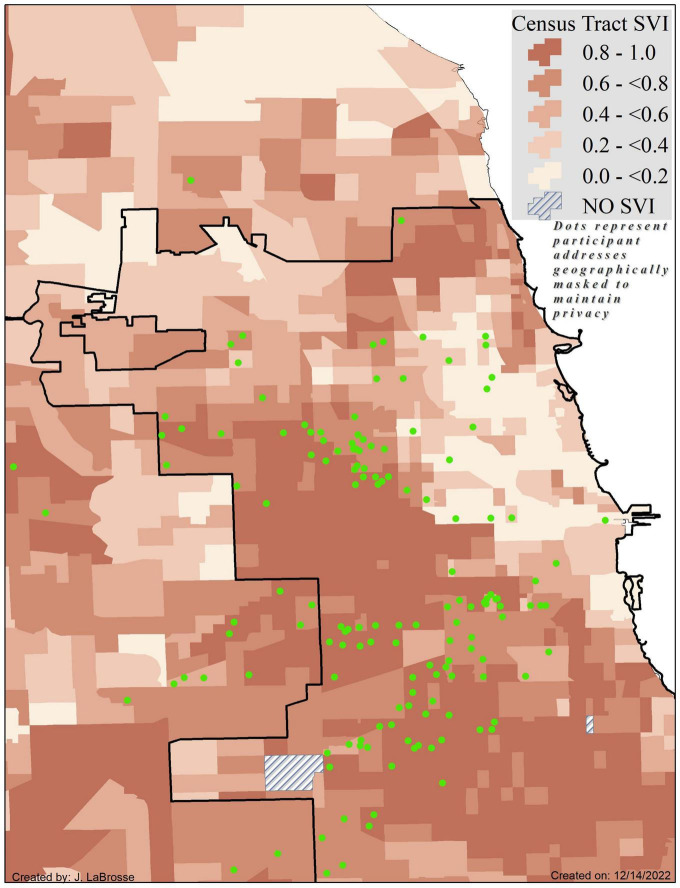
Census tract level Social Vulnerability Index scores by quintiles for the Chicagoland area as measured by the Centers for Disease Control and Prevention with Latino participant locations (green dots) geographically masked to protect confidentiality and maintain privacy. Higher SVI scores and corresponding darker shades of orange signify greater vulnerability. Map coverage is reflective of the geographic center of participant addresses.

Participants (*N* = 780) were on average 72.7 ± 6.3 years of age, primarily (76.9%) female, with approximately 13.9 ± 4.3 years of education. As seen in Table 1, non-Latino Black participants were older than Latino participants and reported more years of formal education (*p*-values < 0.0001). The majority of Latinos (75.2%) preferred to conduct their annual study visits in Spanish. Overall, Latinos lived in more socially vulnerable neighborhoods than non-Latino Black participants, t (778) = −2.3, *p* = 0.018 with Figures 1, 2 providing geographic displays of SVI for non-Latino Black and Latino participants, respectively. Additional information on these and other variables of interest may be found in Table 1 (participant characteristics) and Table 2 (SVI predictor, cognitive and motor outcomes).

**TABLE 1:** Participant characteristics at baseline.

	Non-Latino Black adults	Latino adults	Test statistic
Sample size	590	190	
Age (years)	73.5 (6.3)	70.3 (5.8)	t (778) = 6.1, *p* < 0.0001
Sex (male: female ratio)	451:139	149:41	χ(1) = 0.31, *p* = 0.57
Education (years)	15.1 (3.3)	10.4 (4.9)	t (778) = 14.8, *p* < 0.0001
**Self-reported country of origin (n, %)**			
United States (50 States/DC only)	584, 99.0%	30, 15.8%	–
US Territory of Puerto Rico	–	32, 16.8%	–
Mexico	–	104, 54.7%	–
South America	–	13, 6.8% *Ecuador* = *5*, *Columbia* = *5, Peru* = *3*	–
Central America	2, 0.33% *Honduras* = *1*, *Republic of Central America* = *1*	9, 4.7% *Honduras* = *4*, *Guatemala* = *3, El Salvador* = *1*, *Panama* = *1*	–
Caribbean/Afro-Caribbean	3, 0.50% *Jamaica* = *2*, *Trinidad* = *1*	2, 1.0% *Cuba* = *2*	–
Western Europe	1, 0.17% *Germany* = *1*	–	
Language of testing (n, % Spanish)	0, 0%	143, 75.2%	–

All values are mean (standard deviation) unless otherwise noted; t, t-statistic and χ, Chi-Square statistic.

**TABLE 2:** Neighborhood-level predictor (and themes) as well as individual-level outcomes.

	Non-Latino Black adults	Latino adults
Social vulnerability index	0.64 (0.22)	0.68 (0.23)
**Cognitive functioning**		
Global cognition	0.09 (0.53)	−0.16 (0.58)
Episodic memory	0.09 (0.65)	−0.11 (0.69)
Semantic memory	0.10 (0.76)	−0.09 (0.82)
Working memory	0.18 (0.72)	−0.57 (0.74)
Visuospatial ability	0.03 (0.81)	−0.01 (0.81)
Perceptual Speed	0.06 (0.72)	−0.08 (0.79)
**Motor function**		
Global motor functioning	1.01 (0.17)	0.95 (0.13)
Dexterity	1.00 (0.015)	1.02 (0.14)
Gait	1.03 (0.22)	0.92 (0.15)
Hand strength	1.03 (0.25)	0.88 (0.21)

All values are mean (standard deviation) unless otherwise noted. Given that there are known differences in lived experience between minoritized ethno-racial groups living in the US and it was our study goal to focus on within group heterogeneity, we have chosen not to report between-group differences in Table 2.

### 3.1. SVI and cognitive functioning

Results of demographically-adjusted linear mixed effects models investigating the relationship between baseline SVI and baseline level of and change in global cognitive functioning indicated significant associations for non-Latino Black participants only. More specifically, higher neighborhood-level social vulnerability was associated with lower baseline levels of global cognition for non-Latino Black adults (estimate = −0.19, standard deviation (SD) = 0.09, *p* = 0.029). No such relationship was noted for Latinos (global cognition estimate = 0.03, SD = 0.17, *p* = 0.83). Neither ethno-racial group showed associations between SVI and longitudinal change in cognitive functioning (non-Latino Black participants’ estimate = 0.02, SD = 0.02, *p* = 0.20; Latinos’ estimate = −0.03, SD = 0.04, *p* = 0.52). It should be noted that within the Latino participants, after further adjustments for language preference of testing, SVI and global cognitive results did not change from those reported above (level estimate = 0.05, SD = 0.17, *p* = 0.77; change estimate = −0.03, SD = 0.04, *p* = 0.54).

We further investigated SVI as it related to the five cognitive domains using identical models as described above but substituting global cognition for cognitive domain scores as separate outcomes. For non-Latino Black participants, results (outlined in detail in Table 3A) indicated that higher neighborhood-level social vulnerability was associated with lower baseline levels of episodic memory and slower decline in visuospatial abilities over time (*p*-values <0.015). No relationships between SVI and level of or change in any cognitive domain score were noted for Latinos (Table 3B), even after additional adjustment for language preference of testing (Supplementary Table 1).

**TABLE 3:** Follow-up associations of the Social Vulnerability Index with the five cognitive domains for **(A)** non-Latino Black and **(B)** Latino participants.

(A)					
	Episodic memory	Semantic memory	Working memory	Visuospatial ability	Perceptual speed
SVI	−**0.27 (0.11) *p* = 0.015**	−0.08 (0.13) *p* = 0.51	−0.18 (0.13) *p* = 0.16	−0.19 (0.13) *p* = 0.15	−0.08 (0.11) *p* = 0.456
SVI*time	0.04 (0.02) *p* = 0.12	−0.002 (0.02) *p* = 0.95	0.01 (0.01) *p* = 0.49	**0.04 (0.01) *p* = 0.011**	−0.01 (0.01) *p* = 0.38
**(B)**					
	**Episodic memory**	**Semantic memory**	**Working memory**	**Visuospatial processing**	**Perceptual speed**
SVI	0.11 (0.22) *p* = 0.60	−0.04 (0.24) *p* = 0.86	−0.04 (0.22) *p* = 0.83	0.13 (0.23) *p* = 0.56	−0.20 (0.22) *p* = 0.37
SVI*time	0.03 (0.06) *p* = 0.55	0.04 (0.06) *p* = 0.54	−0.05 (0.07) *p* = 0.44	−0.009 (0.07) *p* = 0.90	0.03 (0.05) *p* = 0.47

Values are unstandardized coefficient (standard error) p-value from linear mixed effects models including additional terms for time (in study) as well as age, sex, education, and interactions (*) of these variables with time. Bolded values denote significance at p < 0.05. Adding language preference for testing (Spanish or English) did not change reported results for older Latinos (see Supplementary Table 1 for details).

### 3.2. SVI and motor functioning

As detailed in Table 4, results of demographically-adjusted linear mixed effects models investigating the relationship between baseline SVI and level of and change in global motor functioning indicated significant level but not change effects for both non-Latino Black and Latino participants. Specifically, higher neighborhood-level social vulnerability was associated with lower baseline global motor functioning (*p*-values <0.020) but not longitudinal change (*p*-values >0.25). We then investigated the SVI index as related to the three motor domains for both non-Latino Black and Latino participants. Results of these analyses (Table 4A) indicated that higher neighborhood-level social vulnerability was associated with lower baseline dexterity and gait performance (*p*-values <0.026) as well as faster decline in hand strength (*p* = 0.012) for non-Latino Black adults. For Latinos (Table 4B), higher neighborhood-level social vulnerability was associated with lower baseline dexterity only (*p* = 0.015). Further adjustments for language preference of testing within analyses involving Latinos did not change reported results (Supplementary Table 2).

**TABLE 4:** Associations of the Social Vulnerability Index with global motor functioning and the three motor domains for **(A)** non-Latino Black and **(B)** Latino participants.

(A)				
	**Motor functioning**	**Hand strength**	**Dexterity**	**Gait**
SVI	−**0.10 (0.03)** ***p* = 0.0008**	−0.05 (0.04) *p* = 0.29	−**0.08 (0.03) *p* = 0.002**	−**0.08 (0.03) *p* = 0.017**
SVI*time	0.004 (0.003) *p* = 0.25	−**0.01 (0.006)** ***p* = 0.012**	0.002 (0.004) *p* = 0.70	0.002 (0.004) *p* = 0.65
**(B)**				
	**Motor functioning**	**Hand strength**	**Dexterity**	**Gait**
SVI	−**0.10 (0.04) *p* = 0.020**	−0.05 (0.06) *p* = 0.43	−**0.11 (0.04) *p* = 0.015**	−0.10 (0.05) *p* = 0.057
SVI*time	−0.008 (0.01) *p* = 0.51	−0.01 (0.02) *p* = 0.48	−0.002 (0.01) *p* = 0.84	−0.01 (0.02) *p* = 0.51

Values are unstandardized coefficient (standard error) p-value from linear mixed effects models including additional terms for time (in study) as well as age, sex, education, and interactions (*) of these variables with time. Bolded values denote significance at p < 0.05. Adding language preference for testing (Spanish or English) did not change reported results for older Latinos (see Supplementary Table 2 for details).

## 4. Discussion

In this study of nearly 800 older non-Latino Black and Latino participants, we investigated a comprehensive measure of neighborhood-level social vulnerability as it related to level of and change in cognitive and motor functioning (and their respective domains) within ethno-racial groups. Results suggested that the SVI was associated with level of as well as change in cognition for older Black adults only such that higher baseline neighborhood-level social vulnerability was associated with lower baseline levels of global cognition (driven primarily by episodic memory performance) and slower declines in visuospatial abilities. In contrast, social vulnerability was associated with motor functioning for both ethno-racial groups. Specifically, within non-Latino Black adults, higher baseline neighborhood-level social vulnerability was associated with lower baseline global motor functioning (driven by dexterity and gait performance) as well as faster rates of decline in strength. For Latinos, higher baseline neighborhood-level social vulnerability was associated with lower baseline global motor functioning driven primarily by dexterity performance. These results revealed differential involvement of neighborhood-level social vulnerability as related to cognitive and motor functioning by ethno-racial group despite the fact that Latinos lived in neighborhoods with greater levels of social vulnerability.

Results of this study contribute to the literature on neighborhood-level health and aging in several ways. First, we extended the use of the Social Vulnerability Index (SVI), previously employed in other areas of aging research including physical activity (An and Xiang, 2015; Armstrong et al., 2015) to the field of cognition showing it may be equally applicable for brain-behavior research. Second, by focusing on non-Latino Black and Latino participants and conducting our analyses stratified by these groups we have contributed specific ethno-racial information to the literature. Third, we expanded previous reports of associations between self-reported participant-level social vulnerability and cross-sectional evaluations of cognitive impairment (Shega et al., 2012) and 5-year cognitive decline (Andrew and Rockwood, 2010), to a study of neighborhood-level determined social vulnerability and cognition as well as motor functioning. Lastly, by considering neighborhood-level vulnerability as related to both cognitive and motor outcomes that may be precursors to ADRD health disparities, we have answered recent calls in the literature for more research on the role of the neighborhood environment in health disparities ADRD research (Hirsch et al., 2022).

Of our two ethno-racial groups of interest, only non-Latino Black participants showed significant associations between neighborhood-levels of social vulnerability and cognition evidencing adverse associations with baseline levels of cognition but positive associations with changes in cognition over time. Our baseline results are in keeping with other research suggesting that those most likely to experience negative associations of an adverse neighborhood milieu on cognition are non-Latino Black adults as opposed to other ethno-racial groups including Latino adults (Besser et al., 2018; Pohl et al., 2021). In fact, the cognitive domain most often reported as negatively associated with an adverse neighborhood environment for non-Latino Black adults is episodic memory (e.g., Meyer et al., 2021; Pohl et al., 2021); see Chen et al. (2022) for a more general systematic review. This was the cognitive domain driving non-Latino Black adults’ significant SVI and global cognition level-based association in the current study as well. In contrast, higher neighborhood-level social vulnerability was also associated with slower decline in visuospatial abilities over time in this same ethno-racial group. Other studies have shown a positive influence of the neighborhood milieu on longitudinal change in cognition (Clarke et al., 2015; Wu et al., 2017; Besser et al., 2021); however, these studies were focused more exclusively on access to resources including public transportation and spaces, retail outlets, and opportunities for social and physical engagement. These resources, while inherent in an urban setting like Chicago (the location of our cohort studies), were not captured by the SVI; however, they are likely correlated with it given that urban environments tend to have greater land use mix including multi-unit structures, street connectivity, and public transportation than more rural areas potentially prohibiting the availability of individualized homes or negating the need for a personal vehicle – both of which would actually increase the SVI score. Furthermore, access to urban resources and the activities they promote, e.g., greater walking destinations and/or social and physical engagement generally, have been shown to facilitate the maintenance or improvement of mental flexibility in older adults (Clarke et al., 2015; Wu et al., 2017; Besser et al., 2021); an aspect of behavior that likely contributes to successful performance on our measures of visuospatial ability (i.e., Progressive Matrices and Judgment of Line Orientation). More work is needed incorporating both neighborhood-level barriers (like those assessed with the SVI) as well as facilitators (like those mentioned as missing from the SVI) of cognitive aging within minoritized communities to fully explore not only the positive but also the null associations between SVI and cognition.

Another facilitator that should be explored, and may help to explain the lack of association between SVI and cognition for our Latino participants despite the fact that they live in more vulnerable neighborhoods than our non-Latino Black participants, is neighborhood ethnic density. Operationally defined in numerous ways including the proportion of a neighborhood comprised of an ethno-racial group either in isolation, or in comparison to other groups, neighborhood ethnic density can be a facilitator of cognitive aging for both non-Latino Black (Pohl et al., 2021) and Latino (Sheffield and Peek, 2009) adults. For example, residence in an immigrant enclave was found to be protective against prevalent cognitive impairment for older foreign-born Mexican Americans (Weden et al., 2017). Neighborhood ethnic density, as well as other Latino-centric lived experience considerations inherent in such enclaves (Weden et al., 2017), may be buffering the adverse effect of the neighborhood exposures represented by the SVI in the current study. While adding a proxy of neighborhood ethnic density to our statistical models did not change results for older Latinos of this study (data not shown), it may be that this variable is only part of a larger framework for how enclave residence and the neighborhood health of the larger environment may interact to impact individual-level cognition, especially for Latinos. While beyond the scope of the current research, future work is needed using a larger suite of variables measuring neighborhood ethnic density and other race- and ethnicity-centric construct (e.g., acculturation in context of Latinos lived experience; Lamar et al., 2023) and more complex statistical techniques (e.g., path analysis) if we are to truly understand the interplay of social vulnerability, neighborhood ethnic density, and cognitive health.

Both non-Latino Black and Latino participants showed significant associations between neighborhood-levels of social vulnerability and global motor functioning; however, associations with motor domains differed by ethno-racial group. In fact, while the negative association between SVI and level of global motor functioning was driven, in part by lower levels of dexterity and gait performance for non-Latino Black adults, dexterity was the sole motor domain of significance for Latinos. Furthermore, only non-Latino Black adults showed a negative association between neighborhood-level social vulnerability and faster declines in motor functioning, specifically hand strength, over time. The SVI as defined by the CDC has been shown to predict older adults’ odds of being physically inactive during their leisure time (An and Xiang, 2015); however, physically activity was based on self-report in that study and it lacked more objective measures of physical and/or motor functioning. Our results suggest that the adverse relationship between neighborhood-level vulnerability and physical activity may extend to frank motor performance including upper *and* lower extremity functioning for non-Latino Black adults. More work is needed to understand these relationships as well as the association between higher levels of neighborhood social vulnerability and faster rates of decline in hand strength also seen in non-Latino Black but not Latino adults.

There are several direct and indirect means by which neighborhood-level social vulnerability may impact cognitive and motor outcomes. Directly, higher neighborhood-levels of social vulnerability may impede an individual’s engagement in cognitively (Clarke et al., 2012, 2015) and/or physically/motorically (An and Xiang, 2015) stimulating activities reducing an individual’s capacities in these areas of functioning. Indirectly, aspects of the neighborhood environment, including some captured by the SVI, have been shown to be associated with a higher likelihood of adverse health outcomes including incident hypertension (Gao et al., 2022) and incident cardiovascular disease (Kershaw et al., 2015). In fact, adverse neighborhood environments have been shown to accelerate cardiometabolic aging and chronic illness in African Americans from the Family and Community Health Study (Lei et al., 2018). These cardio-health conditions are known to negatively impact cognition (Gorelick et al., 2017) including episodic memory (Gonzales et al., 2017), and thought to increase health disparities in cognition (De Anda-Duran et al., 2022) and motor functions (e.g., gait; Niermeyer, 2018 for a systematic review). Additionally, a recent study found that adverse neighborhood conditions are directly related to participant-level psychosocial stress-levels of which negatively impact the Hypothalamic-Pituitary-Adrenal axis in humans (Gerritsen et al., 2010) as well as microglia-dependent mechanisms in rodents (Garvin and Bolton, 2022)-and this stress was in turn related to negative health outcomes (Egede et al., 2022). Thus, it would seem that neighborhood-level social vulnerability gets “under the skin” and “into the brain” of older adults to influence both cognitive and motor outcomes. Future work is needed elucidating direct and indirect mechanisms underlying the associations between SVI and levels of as well as changes in behavior within non-Latino Black and Latino adults.

This study is not without limitations. For example, while the range of behavioral follow-up for non-Latino Black participants reached a maximum of 18 years for cognitive and 16 years for motor assessments, it was less than half that for Latinos and averaged 4 and 3 years, respectively. This may have limited our ability to detect change in cognitive and motor functions for Latinos; annual testing is ongoing and we hope to revisit these analyses once more data has been accrued for this ethno-racial group. We are also actively calculating historic duration of exposure in our cohort studies; however, the lack of this information for the current research-despite the relative stability of the participants included-is nonetheless a limitation. The SVI was estimated using US Census Bureau data obtained from the 2000 and 2010 Census, and more recently, American Community Survey data obtained from 2014, 2016, and 2018; thus, all limitations of this source data are limitations of our data. Additionally, other measures of neighborhood vulnerability or disadvantage exist (e.g., Kind et al., 2014) and have also been used with cognitive outcomes in older adults (Zuelsdorff et al., 2020); however, the SVI has a high degree of construct validity to these measures and, at times, outperforms them in minoritized communities (Ong and Ong, 2020). Lastly, while we adjusted for key demographic characteristics, additional considerations such as nativity status (a potential proxy for neighborhood exposure in early life; Lamar et al., 2020), as well as individual-level socioeconomic status or engagement in cognitive or physical activity were not included as covariates. This is due, in part to the fact that these, and other social determinants of health, may serve as mediators and/or moderators of our relationships of interest and deserve more in-depth study that is beyond the scope of this manuscript.

Strengths of this work should also be highlighted. First, our comprehensive approach to cognitive and motor functioning ensured that we were able to investigate not only global performance but multiple domains of functioning for each behavioral construct. Further, by analyzing our data within ethno-racial groups, we were able to better understand neighborhood-level factors associated with specific ethno-racial cognitive and motor aging trajectories. Lastly, our Latino cohort was relatively diverse including individuals from across Mexico, Central and South America, as well as the Caribbean, although, we did not have adequate representation to investigate potential differences in our results by country of origin (e.g., only 2 Cuban participants). In sum, results of this study suggest that the neighborhood-level milieu, as measured by the SVI, may have more of an impact on cognition for older non-Latino Black adults, but may be equally important for motor functioning among both non-Latino Black adults and Latinos. Future study is needed to confirm these results and explore relevant neighborhood-level targets for intervention within each ethno-racial group.

## Data availability statement

Publicly available datasets were analyzed in this study. This data can be found here: Rush Alzheimer’s Disease Center (RADC) Research Resource Sharing Hub (www.radc.rush.edu).

## Ethics statement

The studies involving human participants were reviewed and approved by the Rush University Medical Center. The patients/participants provided their written informed consent to participate in this study.

## Author contributions

ML and LB contributed to the conception and design of the study. ML and BL-M organized the database. ML and SL performed the statistical analysis. ML wrote the first draft of the manuscript. All authors contributed to manuscript revision, read, and approved the submitted version.
