# Supplementary material for: Neighborhood-level social vulnerability and individual-level cognitive and motor functioning over time in older non-Latino Black and Latino adults
Source: Front Hum Neurosci. 2023 May 12;17:1125906. doi: 10.3389/fnhum.2023.1125906 (PMC10213534; doi:10.3389/fnhum.2023.1125906)
Supplement: Supplementary file 1 [file Table_1.DOCX]

Supplementary Material

Neighborhood-level social vulnerability and individual-level cognitive and motor functioning over time in older non-Latino Black and Latino adults

**Melissa Lamar, Ph.D.^1,2^, Kiarri N. Kershaw, Ph.D. ^3^, Sue E. Leurgans, Ph.D.^1,4^, R. Reshmi Mukherjee^1,5^, Brittney Lange-Maia, Ph.D.^1,6^, David X. Marquez, Ph.D.^1,7^ & ^1,2,4^Lisa L. Barnes, Ph.D.**

*** Correspondence:** Melissa Lamar: [melissa_lamar@rush.edu](mailto:melissa_lamar@rush.edu)

**SUPPLEMENTARY TABLE 1.** Associations of the Social Vulnerability Index with the five cognitive domains for Latino participants further adjusting for language preference for testing

|  | Episodic Memory | Semantic Memory | Working Memory | Visuospatial Processing | Perceptual Speed |
| --- | --- | --- | --- | --- | --- |
| SVI | 0.13 (0.21) p=0.54 | -0.02 (0.24) p=0.91 | -0.02 (0.21)  p=0.92 | 0.17 (0.23) p=0.47 | -0.19 (0.22) p=0.40 |
| SVI*time | 0.03 (0.06) p=0.55 | 0.04 (0.06) p=0.52 | -0.05 (0.07)  p=0.46 | -0.01 (0.07) p=0.88 | 0.03 (0.05) p=0.47 |

*Note.* Values are unstandardized coefficient (standard error, p-value) from linear mixed effects models including additional terms for time (in study) as well as age, sex, education, language preference for testing and interactions of these variables with time.

**SUPPLEMENTARY TABLE 2.** Associations of the Social Vulnerability Index with global motor functioning and the three motor domains for Latino participants further adjusting for language preference for testing

|  | Motor Functioning | Hand Strength | Dexterity | Gait |
| --- | --- | --- | --- | --- |
| SVI | **-0.10 (0.04)**  **p=0.022** | -0.05 (0.06)  p=0.44 | **-0.11 (0.04) p=0.018** | -0.09 (0.05) p=0.065 |
| SVI*time | -0.008 (0.01)  p=0.52 | -0.01 (0.02)  p=0.48 | -0.002 (0.01) p=0.84 | -0.01 (0.02) p=0.52 |

*Note.* Values are unstandardized coefficient (standard error, p-value) from linear mixed effects models including additional terms for time (in study) as well as age, sex, education, language preference for testing and interactions of these variables with time. Bolded values denote significance at p<0.05.
